# *Rubus ulmifolius* Schott as a Novel Source of Food Colorant: Extraction Optimization of Coloring Pigments and Incorporation in a Bakery Product

**DOI:** 10.3390/molecules24112181

**Published:** 2019-06-10

**Authors:** Liliana Primo da Silva, Eliana Pereira, Miguel A. Prieto, Jesus Simal-Gandara, Tânia C.S.P. Pires, Maria José Alves, Ricardo Calhelha, Lillian Barros, Isabel C.F.R. Ferreira

**Affiliations:** 1 Centro de Investigação de Montanha (CIMO), Instituto Politécnico de Bragança, Campus de Santa Apolónia, 5300-253 Bragança, Portugal; lili.262@hotmail.com (L.P.d.S.); eliana@ipb.pt (E.P.); tania.pires@ipb.pt (T.C.S.P.P.); maria.alves@ipb.pt (M.J.A.); calhelha@ipb.pt (R.C.); 2 Nutrition and Bromatology Group, Faculty of Food Science and Technology, University of Vigo, Ourense Campus, E32004 Ourense, Spain; mprieto@ipb.pt (M.A.P.); jsimal@uvigo.es (J.S.-G.)

**Keywords:** *Rubus ulmifolius* Schott fruits, natural colorant, anthocyanins, heat assisted extraction, response surface methodology, food incorporation

## Abstract

(1) Background: Color has been considered to be the flashiest attribute of foodstuffs and researchers have shown a great interest in the extraction of pigmented compounds from vegetal products, with the purpose to provide alternative counterparts to the food industry; (2) Methods: This study aimed to explore *Rubus ulmifolius* Schott fruits as a potential source of anthocyanins, optimizing the extraction method, evaluating the bioactivity and incorporating the rich extract into a bakery food product; (3) Results: After the extraction optimization, results showed *R. ulmifolius* fruits to be a great source of anthocyanins, obtaining an amount of 33.58 mg AT/g E, with an extraction yield of 62.08%. The rich anthocyanin extract showed antitumor and antimicrobial potential in some tumor cell lines and strains, respectively, as well as the absence of toxicity; (4) Conclusions: The extract when incorporated in a bakery product showed a good coloring capacity, maintaining the nutritional value, revealing its use to be a great approach for replacing artificial colorants.

## 1. Introduction

Color is a parameter with enormous relevance for the food industry, being considered the oldest criterion used by consumers to judge the quality of food products [1,2]. Thus, colorants have been used by the food industry for different proposes, namely, to increase the organoleptic value and enhance the natural color [2,3].

In recent years, the scientific community have carried out several studies, with the propose to obtain pigmented compounds (from natural matrices) and incorporate them in the food industry and, thus, to guarantee higher levels of quality and safety, compared to artificial colorants (which have been revealing a negative impact in human health [4,5].

Anthocyanins (E163) are a group of phenolic compounds and have been the first choice for producers to color foodstuff [6], due to their pigmentation potential and their extraordinary water solubility, which allows easier incorporation into aqueous food systems [7].

Several methodologies have been used to optimize the extraction of these pigments, with some examples being heat assisted extraction (HAE) and also methods coupled to green technological advances, including ultrasound-assisted extraction (UAE), microwave-assisted extraction (MAE), supercritical fluid extraction (SFE), accelerated solvent extraction (ASE), pressurized liquid extraction (PLE) and others [8,9]. HAE (a maceration technic) is a solid-liquid extraction method which consists involves mixing solids with a solvent under specific conditions (temperature and time) and continuous electro-magnetic stirring. HAE is considered to be a convenient approach on a laboratorial scale because it is a simple technique with low requirements in terms of equipment, and, consequently, lower financial costs [10,11].

However, the extracted compounds are susceptible to degradation and the stability depends on several factors, namely, external influences such as: pH, temperature and light [12,13]. The pH is the principal impact in color stability, due to the occurring structural changes. In this sense, the color shows the highest stability at pH 1 in which the flavylium cation form is predominant (red); at values between pH 2 and 4 it presents the blue quinoid bases form (red to blue); whereas, above pH 5, due to a nucleophilic attack by water, the carbinol pseudo bases and the chalcones, which are both unstable and colorless, are formed [14,15].

In addition to the color attributes, the interest in anthocyanins has intensified due to their therapeutic activities, which include anti-inflammatory, anti-carcinogenic, antimicrobial [12,16], and antioxidant activity. Antioxidant activity is particularly notable because it intervenes in the prevention of biomolecules oxidation (such as lipids and proteins), cardiovascular and neuronal diseases, cancer and diabetes [1,17]. *Rubus ulmifolius* Schott fruits could be considered a good source of anthocyanin compounds. This specie belongs to the *Roseaceae* family and is a perennial shrub, widely distributed in Europe, well-known for its edible fruits (blackberries). These fruits are very appreciated by consumers for its color, flavor, taste and also for the rich composition in bioactive compounds [13,18,19]. Thus, the present study aims to perform the extraction of natural food colorants (anthocyanins) from *Rubus ulmifolius* Schott fruits. For this propose, an optimization of the extraction method (HAE) was carried out using the response surface methodology (RSM), followed by bioactivity studies of the extract rich in anthocyanin compounds, for further incorporation into a bakery food product.

## 2. Results and Discussion

### 2.1. Extraction Optimization by RSM Method

Although there are abundant studies relating to the extraction of anthocyanins from natural sources [6,10,11,20], studies finding optimal conditions for maximizing the extraction of anthocyanins from *R. ulmifolius* fruits are absent. There is a great diversity of anthocyanin composition among species, with some discrepancies being found between the same species which could be attributed to a wide range of factors as plant genetics, growing conditions (e.g., climate conditions), plant physiology (e.g., state of ripeness) and different parts of the plant (e.g., fruit and leaf) [15]. Moreover, the extraction, identification and quantification methods have been considered as relevant factors. Thus, it is pertinent to individualize each study in order to find proper conditions and extraction methods to achieve the highest yields [9]. The first approach of this study was to optimize the efficiency of HAE to recover anthocyanins from *R. ulmifolius* fruits through the application of an RSM technique. RSM is a technical procedure that allows us to detect the patterns of multiple variables and the possible complex interactions [21]. Additionally, RSM easily permits us to find optimal conditions that maximize or minimize such responses. To accomplish these objectives, there are several experimental designs available, each of them with different advantages and disadvantages, but in general, its application significantly reduces the number of experimental runs needed. In this regard, the *CCCD* with five levels per factor is a popular experimental design for RSM and has been applied by a number of researchers in the optimization of various food processing methods [22,23]. Therefore, for optimization purposes, the RSM experimental design of *CCCD* with five levels of variation for the three independent variables was used for optimizing the conditions of anthocyanin extraction using the following variables and associated ranges: *t* (20−120 min), *T* (20−90 °C) and *S* (0−100%). The response results used to optimize the anthocyanin extraction were the anthocyanin content (either the total amount or the four individual anthocyanins) and the extraction yield according to the *CCCD* design, and the results are described in part A of Table 1.

A detailed description of the coded and natural values of the selected variables for HAE used in *CCCD* design is presented in Table 2.

The yield of the extracted material ranged from 40.44% to 74.03%. As described (in Section 3.4), the anthocyanin compound A2 (cyanidin-3-*O*-glucoside) dominated the other ones (A3 to A5), with levels ranging from 2.91 to 24.06 mg/g E. For the other compounds the ranges oscillated as follows: A3 (pelargonidin-3-*O*-glucoside, from 1.03 to 3.09 mg/g E), A4 (cyanidin-3-*O*-xyloside, from 1.15 to 6.45 mg/g E) and A5 (cyanidin-3-*O*-dioxayl-glucoside, from 0.56 to 2.16 mg/g E). Finally, the values of the total anthocyanin content (AT) varied from 5.87 to 35.55 mg/g E.

The different response criteria used presents a major advantage for industrial sectors, which can recover high added-value compounds from plant materials and use them as natural colorants or other bio-based ingredients. Taking this into consideration, the response criteria applied are particularly important for providing information about the amount of plant material needed to obtain a certain quantity of the target compounds, and the concentration of these compounds in the obtained extracts.

The parametric values of the second-order polynomial model of Equation (1) obtained after fitting the extraction response format values and the corresponding statistical information (α = 0.05) are presented in part B1 of Table 1. The fitting procedure of Equation (1) applied to the experimental responses was performed using nonlinear least-squares estimations and those that had non-significant (ns) values were excluded.

**Figure 1:**
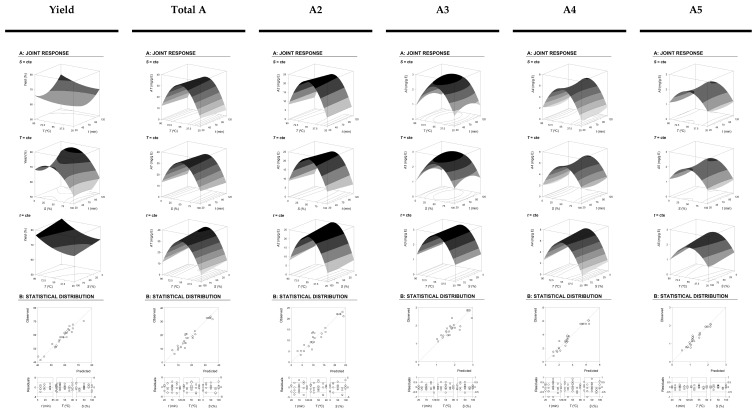
Shows the HAE graphical results for the extraction yield of the extracted (%) material obtained, the total anthocyanin content (AT, mg AT/g E), the major anthocyanin compound detected (A2, mg A2/g E) and three minor anthocyanin compounds detected (A3 to A5, in mg/g E). Each figure is divided in two parts. *Part A*: Shows the graphical analysis by net surfaces that represents the 3D response surface predicted with the second order polynomial of Equation (1). The binary actions between variables are presented when the excluded variable is positioned at the individual optimum (Table 1B2). The experimental design and results are described in Table 1**A**. *Part B*: To illustrate the goodness of fit, two basic graphical statistic criteria are used. The first one is the ability to simulate the changes of the response between the predicted and observed data; and the second one is the residual distribution as a function of each of the variables.

The developed models (Table 1 part B1) can be used to determine the absolute/relative optimal values of the variable conditions to maximize the responses individually and globally in order to obtain the most efficient extraction. Part B2 of Table 1 shows the HAE individual and global optimal response values and the corresponding conditions for the responses assessed. Although the parametric values show the responses and can be used to understand the patterns of the responses, the best way to express the effects of any independent variable on the extraction of any type of response is to generate 3D surface plots, varying two variables in the experimental range under investigation and holding the other two variables at their fixed level. In this regard, Figure 1 shows the 3D surface plots parameters on the extraction behavior. The plots enable us to visualize the influence and interaction between the variables. Visual analysis of 3D surface and contour plots are in accordance with parametric values derived from the multiple regression analysis described in part B1 of Table 1. The following section describes the response patterns.

The extraction results of HAE are a function of the combination of the three main variables involved (*X*_1–3_: t, *T*, and *S*) can be observed in Figure 1. In a more detailed form, part A of all figures in Figure 1 shows the 3D surface plots of the extraction obtained (Yield, %), the total anthocyanin content (AT, mg AT/g E), the major anthocyanin compound detected (A2, mg A2/g E), and the three minor anthocyanin compounds detected (A3 to A5, in mg/g E). These graphical illustrations are helpful to visualize the tendencies of each response and guide the selection of the most favorable conditions, while simultaneously considering all responses.

Traditionally, part B of Figure 1 illustrates the capability to predict the obtained results and the residual distribution as a function of each of the considered variables. Regarding statistical terms, the distribution of residues (Figure 1) presents, for the majority, more than 90% reliability, showing good agreement between experimental and predictive values. This is also verified by the high values of *R*^2^ (0.95), indicating the percentage of variability explained by the model.

#### 2.1.1. Numerical Optimal Conditions that Maximize the Extraction and Experimental Verification of Predictive Models

**Figure 2:**
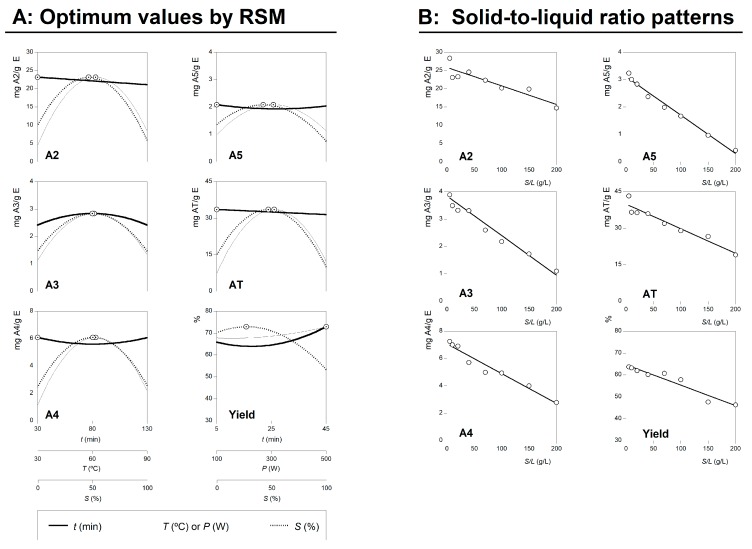
Final summary of the HAE effects of all variables assessed. Part A: Shows the individual 2D responses of all studied responses as a function of all the variables assessed. The variables in each of the 2D graphs were positioned at the individual optimal values of the others (Table 1B2). The dots (⊙) presented alongside each line highlight the location of the optimum value. Lines and dots are generated by the respective theoretical second order polynomial derived from Equation (1). Part B: Shows the dose response of S/L at the global optimal values of the other three variables (Table 1B2). Experimental results are the dots (◯), meanwhile the lines are the predicted pattern created by a linear equation with an intercept. The limit value (~200 g/L) shows the maximum achievable experimental concentration until the sample cannot be physically stirred at laboratory scale.

Based on the experimental results and statistical analysis, numerical optimizations have been conducted in order to establish the optimum level of the independent variables with desirable response levels. In order to verify the predictive mathematical model of the investigated process, the experimental confirmation was performed on the estimated optimal conditions. The predicted results matched with the experimental results obtained at optimal extraction conditions, which were validated by the RSM model with good correlation. The values of the variable conditions that lead to optimal response values for RSM using a *CCCD* are shown in Table 1 and Figure 2 (part A). These values were obtained by combining the information produced by all the responses assessed. This table shows the individual and global optimal variable conditions for HAE and the respective amounts of the extracted anthocyanins.

#### 2.1.2. Dose-Response Analysis of the Solid-to-Liquid Ratio Effect at the Optimal Conditions

The study of *S/L* was performed in the optimal conditions predicted by the RSM models obtained for each response factor (part B2 of Table 1). The individual *S/L* study was designed to verify the behavior between 5 to 200 g/L. The maximum value of 200 g/L was used as the limit condition due to the impossibility of producing a homogenized reaction when higher values were introduced.

The dose responses of the *S/L* obtained were consistent with the results obtained in the RSM analysis, and could be described by a simple linear relationship (shown in Part B of Figure 2). All experimental points are distributed around the equation with only one independent variable and, consequently, the dose response is explained by the slope (*m*) of the linear relation. None of the cases showed positive values of *m* (the extraction efficiency increases as the *S/L* increases), while the parametric value of m showed negative values in all the cases (the efficiency decreases as the *S/L* increases). The parametric values of each individual compounds were: to A2 b = 25.851 ± 2.161 mg/g E, m = −0.051 ± 0.009 and *R*^2^ = 0.9216; to A3 b = 3.840 ± 0.521 mg/g E, m = −0.014 ± 0.004 and *R*^2^ = 0.9434; to A4 b = 7.067 ± 1.262 mg/g E, m = −0.022 ± 0.011 and *R*^2^ = 0.9556; and to A5 b = 3.107 ± 2.161 mg/g E, m = −0.014 ± 0.009 and *R*^2^ = 0.9846. The parameters values of the total anthocyanin content (AT = A2 + A3 + A4 + A5) were: b = 39.864 ± 4.334 mg/g E, m = −0.101 ± 0.022 and *R*^2^ = 0.9284. The parametric values of the extraction yield (%) were: b = 64.606 ± 4.178 mg/g E, m = −0.093 ± 0.001 and *R*^2^ = 0.9273.

Although the initial values of *S/L* lead to similar results, extraction efficiency decreases as the *S/L* ratio increases. Negative values of m show that the highest ratio of *S/L* (200 g/L) results in a decrease of extraction capacity, whereas the lowest ratio (5 g/L) facilitates a more efficient extraction.

To reduce the extraction time and solvent consumption are some of the desired requirements when designing novel extraction methods. The solvent volume should be sufficient only to dissolve the target compounds and promote mass transfer. At industrial scale, higher S/L are desirable to maximize the extraction yield with minimal solvent consumption, thus making the process more productive and sustainable. The extraction rate is also affected by the mass transfer resistance associated with the matrix structure. As shown, the ability of extraction is strongly dependent on the *S/L* ratio, as an increase of 1 g/L leads to a significant reduction of the anthocyanin content at levels of ~45% with *S/L* of 200 g/L, comparatively to 5 g/L. Considering the process of optimization, the ideal value of *S/L* is approximately 25 g/L.

### 2.2. Coloring Potential, Cytotoxicity and Antimicrobial Activity of the Rich Anthocyanin Extract

The rich anthocyanin extract (RAE) was prepared using the conditions previously determined (Section 3.7) that maximize the response (Table 1B1). For further analysis of the coloring potential, bioactivities and incorporation in a bakery product were assayed. Following this procedure, it was obtained a total content of anthocyanin compounds of 33 ± 1 mg AT/g E (Table 2), being similar to the value obtained by the RSM method. Concerning the color parameter (Table 2), it was evident a L* value of 24.8 ± 0.1, a a* value of 31.7 ± 0.5 and a b* value of 7.6 ± 0.2, presenting a red-burgundy color when converted to RGB values (Table 3).

Concerning the bioactive evaluation, the results of antimicrobial activity and cytotoxicity of the RAE are presented in Table 4.

Regarding antimicrobial potential, the RAE exhibited a bacteriostatic effect against all the tested Gram-positive strains, with MIC values ranging between 2.5 and 10 mg/mL. Likewise, against the Gram-negative strains, the RAE also presented activity, namely, against the strains *Escherichia coli*, *Klebsiella pneumoniae*, *Morganella morganii* and *Proteus mirabilis*, with MIC values ranging between 2.5 and 20 mg/mL, while revealing itself to be more efficient against *Morganella morganii* (MIC = 2.5 mg/mL) what was more active than ampicillin (MIC = 20 mg/mL). Several studies have been focused on the search for plant derived extracts, which have been proven to have strong biologic antimicrobial, antitumor, and antioxidant potential, as well as other advantages, due to the presence of phytochemicals, especially flavonoids such as anthocyanins, phenolic acids, alkaloids, and glycoside derivatives [24,25,26]. Many of the bioactive properties present in these natural extracts are mostly available due to synergic mechanisms displayed among the different compounds. Concerning the antibacterial activity, Pertuzatti et al. [25] verified that the polyphenol compounds have a potent effect due to the presence of hydroxyl groups in the phenolic ring that allow them to destabilize the lipid bilayer of bacterial cell membrane, causing structural and functional damage, achieving a strong bacteriostatic activity. Besides that, other mechanisms that are not totally elucidated but that are frequently referred to, are the effects on membrane permeability or inhibition on glucose uptake [27,28]. Also, Sun et al. [29], described a potential antimicrobial mechanism obtained from anthocyanins, due to their possible entrance into the inner membrane, lowering the activity of several enzymes, such as AKP, ATPase and SOD, thus leading to the inhibiting of bacteria growth. At this point, it seems that the extraction optimization process of anthocyanin compounds is the key enabler for the antibacterial effect shown by the RAE as it allowed us to obtain a higher concentration in anthocyanins, thus it did not show any bactericidal effect.

The cytotoxicity of the extracts was evaluated using four malignant cell lines (MCF-7, NCI-H460, HeLa and HepG2) and one primary non-tumor cell culture (PLP2) derived from a porcine liver and the results are also given in Table 4.

In general, the RAE showed an anti-proliferative effect against all the tested tumor cell lines. Despite the similar values obtained, HepG2 cell line revealed to have the highest sensitivity to the RAE with a GI_50_ value of 286 ± 13 µg/mL, and the NCI H460 cell line was more resistant to the RAE, presenting a GI_50_ value of 337 ± 11 µg/mL. The high GI_50_ values means that it needs to apply a larger amount of extract to obtain the inhibition of cell proliferation. However, it is necessary to consider that we are testing an extract and not a pure compound or a mixture of pure compounds. Thus, reporting that the extracts are active against the cell lines under study means that they had the ability to inhibit cell proliferation. The hepatotoxicity evaluation was performed to guarantee the absence of toxicity from the extract, and the analysis did not show any cytotoxicity (PLP2; GI_50_ > 400 μg/mL). Likewise, the activity expressed by the extract could be related to the high concentration of phenolic compounds, namely anthocyanins (33.2 mg AT/g E), which are known for their potent antioxidant activity [30,31], which have a major role in preventing several diseases [29,32]. Dellai et al. [33] described that phenolic compounds have the capacity to inhibit cancer cells through the xenobiotic metabolizing enzymes that modify metabolic activation of several potential carcinogens, and also, by interfering with hormone production and inhibiting the aromatic enzyme, which allows cell cycle progression to be inhibited. Besides that, other mechanisms referred to by these authors are the disruption of cellular division on mitosis, the reduction of essential cellular proteins and colony formation during cancer cell proliferation.

Afterwards, the rich anthocyanin extract was incorporated into a bakery product. Among all the bakery products in the food industry, donut samples were chosen because they are a well-known and appreciated product worldwide. Thus, the replacement of artificial colorants with natural ones could be a strategy for innovation in the food industry and the promotion of healthier food products. Concerning visual appearance, Table 5 presents the results for the different color parameters (CIE L*a*b*) of the donut samples.

The analysis during T0 storage time revealed some statistically significant differences (*p* < 0.05) in this parameter, namely, between the donuts without the RAE (DCT0) and the donuts incorporated with the RAE (DRAET0), meaning that the extract had provided color to the donut sample. The highest difference was verified for the luminosity (L*), obtaining a value of 76 ± 1 for DCT0 sample and 57.5 ± 0.4 for DRAET0, followed by a* parameter with values of −0.1 ± 0.2 and 10.8 ± 0.4, respectively, and for b* a value of 19.7 ± 0.3 and 10.9 ± 0.5, respectively. Concerning the red color of the extract, the difference found between the parameters were predictable, especially for a* parameter, which presents the red color intensity. The differences founded between the DCT0 and DRAET0 are similar to the differences determined between the samples DCT3 and DRAET3 with a value of 77.0 ± 0.3 and 57.0 ± 0.3 for L* parameter, 0.03 ± 0.0 and 10.2 ± 0.1 for a* parameter and 20.0 ± 0.3 and 10.4 ± 0.6 for b* parameter, respectively. After 3 days of storage time no significant differences were found between the control samples (DRAET0 and DRAET3) and between the samples incorporated with RAE (DRAET0 and DRAET3), meaning that the donut color remained unchanged along with the storage time.

Table 5 also shows the results for nutritional and chemical analysis of the donut samples (DC and DRAE). Concerning the nutritional value, carbohydrates were the most abundant macronutrient, followed by proteins, fat, and ash content. Overall, there were only statistically differences (*p* < 0.05) in moisture, carbohydrates and total energy between the samples DCT0 and DRAET0 and between samples DCT3 and DRAET3, which means that the incorporation of the RAE only affected these parameters. For energy, the values ranges between 307.2 ± 0.4 and 332.8 ± 0.4 kcal/100 g fw were expected values for a bakery product.

In the present study, the pH value (Table 5) is an important parameter to control, due to the stability of anthocyanin, thus, the pH was measured in the control donut mixture before baking, with a value of 6.4 ± 0.1, and after RAE incorporation (5.23 ± 0.02). The decrease in the pH value is due to the presence of lemon juice, an ingredient used to dissolve and stabilize the anthocyanin pigments.

The free sugars composition is also described in Table 5. As previously mentioned, the carbohydrates are present in the highest amounts and are mostly represented by free sugars, with a total concentration of 18.1 ± 0.3 (DRAET3) to 19.4 ± 0.7 g/100 g fw (DCT3), with a high prevalence of sucrose, followed by trehalose. Fructose and glucose were detected in the lowest concentration in all samples. Overall, significant differences were detected for all detected sugars between the DC and DRAE for both storage time (T0 and T3). The DRAE always showed the highest amounts of fructose, glucose, and threalose when compared to the DC, which could be explained as being due to the sugars content present in the RAE (data not shown).

Regarding the fatty acids profile, twenty-three fatty acids were identified, with linoleic (C18:2n6) and oleic acid (C18:1n9) being the most abundant molecules. Unsaturated fatty acids prevailed over saturated fatty acid, with the prevalence of polyunsaturated fatty acids (ranging from 60.9 ± 0.1% in DRAET0 to 62.3 ± 0.3 % in DCT3), followed by monounsaturated (22.8 ± 0.6% in DRAET0 to 23.66 ± 0.02 % in DRAET3). Statistical significant differences (p < 0.05) were only found between the DCT3 and DRAET3 samples relatively to PUFA content. Moreover, the storage time did not influence the fatty acid profile as also the percentages of each FAME. A rich fatty acid composition was expected, since several lipid ingredients were used in the donut formulation. Besides that, as the RAE was added in a low amount to provide the desired color, it would not be expected that a high difference would occur between the two types of donut formulations (DRAE and DC).

## 3. Material and Methods

### 3.1. Samples

**Figure 3:**
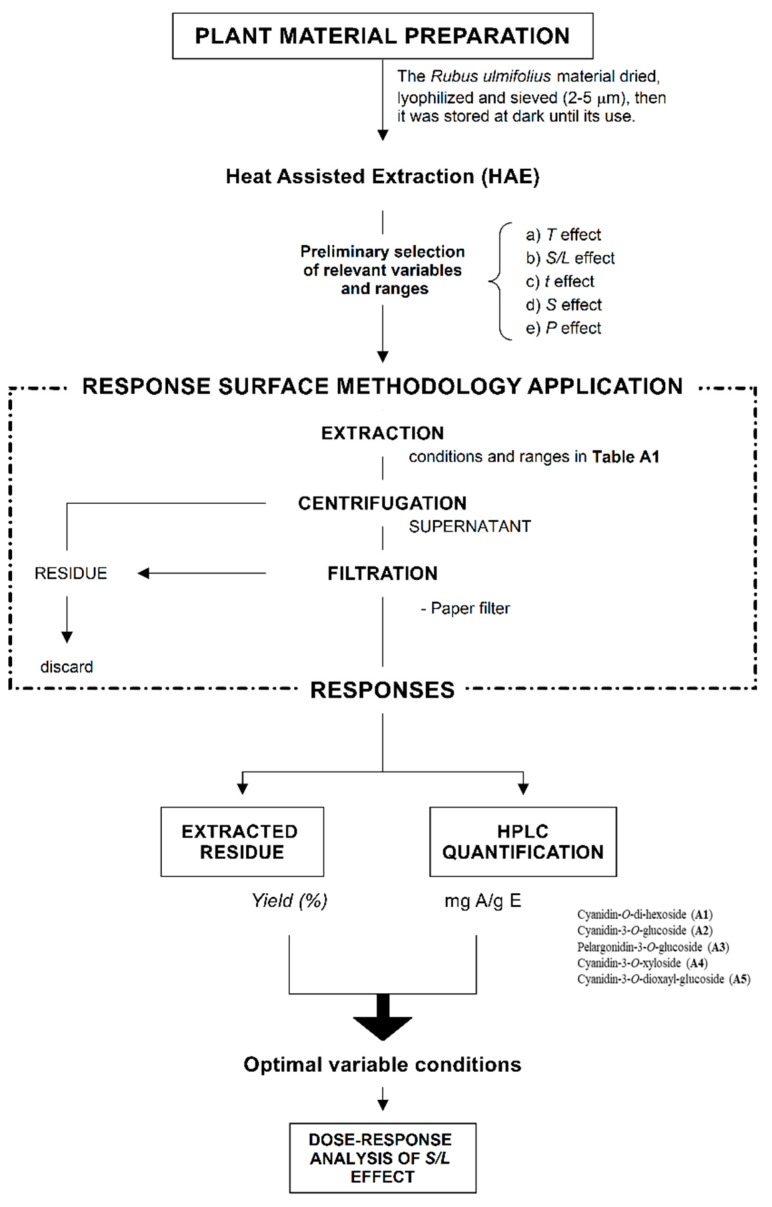
Diagram of the different steps carried out for optimizing the conditions that maximize the extraction responses.

*Rubus ulmifolius* Schott fruits (commonly known as elm-leaf blackberry or wild blackberry) belongs to the *Rosaceae* family, was collected during September 2017 in Bragança, Portugal. The obtained fruits (200 g) were dehydrated by lyophilisation (FreeZone 4.5, Labconco, Kansas City, MO, USA), reduced to a fine dried and homogenous power (~20 mesh) and stored in a suitable location, as described by da Silva et al. (2019). The procedure described is summarized here in Figure 3.

### 3.2. Extraction Technique

Heat assisted extraction (HAE) was performed according to a methodology previously described by Pinela et al. [9], using 1.0 g of the *R. ulmifolius* fruit with 20 mL of solvent with different conditions previously defined by the RSM design (Table 1): time (*t* or *X1*, 20 to 120 min), temperature (*T* or *X2*, 20 to 90 °C) and ethanol proportions acidified with citric acid (pH 2.5) (*S* or *X3*, 0 to 100%). The solid-to-liquid ratio (*S/L* or *X4*) was maintained at 50 g/L. After the extraction, the samples were centrifuged (6000 rpm for 20 min at 10 °C) and filtered (paper filter Whatman nº 4) and the supernatant was collected and divided into two portions for HPLC-DAD and extraction yield analysis. A portion of 3 mL was filtered through a LC filter disk (nylon filter 0.2 µm, 25 mm diameter, Whatman™, GE Healthcare, Buckinghamshire, UK) for chromatographic analysis and another portion of 5 mL was dried in an incubator (Jouan, Berlim, Alemanha) at 105 °C over 48 h for suspended solids determination in order to determine the extraction yield assessment.

### 3.3. Identification and Quantification of Anthocyanin Compounds

The obtained extracts were analyzed by an HPLC-DAD-ESI/MSn system (Dionex Ultimate 3000 UPLC, Thermo Scientific, Waltham, MA, USA) equipped with a C18 reverse phase column AQUA^®^ (5 µm, 150 mm × 4.6 mm i.d, Phenomenex, Torrance, California, EUA), following a procedure previously described by López et al. [11]. Detection was achieved using a DAD (520 nm as the preferred wavelength) and in mass spectrometer (Linear Ion Trap LTQ XL, Thermo Finnigan, San Jose, CA, USA) equipped with an ESI source, operating in positive mode. The anthocyanin compounds were characterized according to their UV–Vis, mass spectra and their retention times, compared with authentic standards (cyanidin-3-*O*-glucoside, y = 104478x − 823429; *R*^2^ = 0.993; pelargonidin-3-*O*-glucoside, y = 50652x − 696848; *R*^2^ = 0.998) and data provided by the literature. A total of five anthocyanin compounds were found and identified previously by da Silva et al. [34].

For quantitative analysis, calibration curves were obtained by injection of known concentrations (200–0.25 μg/mL) of cyanidin-3-*O*-glucoside (y = 104478x − 823429; *R*^2^ = 0.993) and pelargonidin-3-*O*-glucoside (y = 50652x − 696848; *R*^2^ = 0.998).

### 3.4. Response Format Values for the Results Presentation

**Figure 4:**
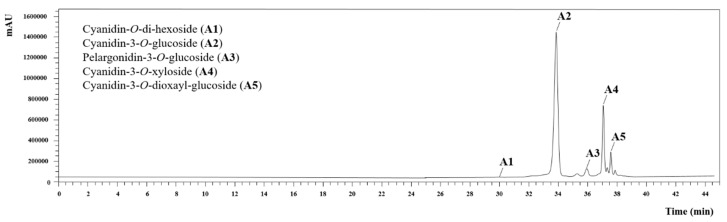
Example of HPLC results regarding the anthocyanin compounds found in analyzed samples.

The individual contents of the four anthocyanin compounds (A2, A3, A4 and A5), total anthocyanins (TA) and the yield of extraction were defined as the responses to be quantified. Five anthocyanin compounds were detected: cyanidin-*O*-di-hexoside (A1), cyanidin-3-*O*-glucoside (A2), pelargonidin-3-*O*-glucoside (A3), cyanidin-3-*O*-xyloside (A4) and cyanidin-3-*O*-dioxayl-glucoside (A5) (Figure 4). However, as A1 was detected in low concentrations, it was not included in the RSM model. The results were expressed as mg of anthocyanin (A or AT) per gram of extract (mg A/g E).

### 3.5. Experimental Design, Model Analysis and Statistical Evaluation

#### 3.5.1. RSM Experimental Design

Trials based on one-at-the-time analysis of each of the variables were conducted. The variables that caused significant changes and the relevant ranges of action were selected (Table 1). The combined effects of these three variables were studied using a circumscribed central composite design (*CCCD*), with five levels for each one [35] and twenty-eight response combinations.

#### 3.5.2. Mathematical Model

The RSM data were calculated as described by Pinela et al. [9], using the equation:

(1) $$y=b_{0}+\overset{n}{\underset{i=1}{\sum }}b_{i}X_{i}+\overset{n-1}{\underset{\begin{array}{c} i=1\\ j>i \end{array}}{\sum }}\overset{n}{\underset{j=2}{\sum }}b_{ij}X_{i}X_{j}+\overset{n}{\underset{i=1}{\sum }}b_{ii}X_{i}^{2}$$

where: *Y*—dependent variable (response variable) to be modelled; *X_i_* and *X_j_* independent variables; *b_0_*—constant coefficient; *b_i_*—coefficient that describes linear individual effect of each variable; *b_ij_*—coefficient responsible for describing the linear interactive mechanisms between two variables; *b_ii_*—coefficient responsible of quadratic effect of each variable; *b_iijj_*—coefficient responsible for describing the quadratic interactive mechanisms between two variables and n is the number of variables. The responses (*Y*) used correspond to extraction yield (Yield, %) and the individual anthocyanin content of the major compounds (A2 to A4) and the total sum (AT).

#### 3.5.3. Procedure to Optimize the Variables to a Maximum Response

A *simplex* method was applied with the objective of optimizing the predictive model by solving nonlinear problems and maximizing the extraction yield and the recovery of phenolic compounds [36]. In this method certain limitations were imposed, such as, times lower than 0; in order to avoid variables with unnatural and unrealistic physical conditions.

#### 3.5.4. Dose-Response Analysis of the Solid to Liquid Ratio

Once the optimal conditions were found and are represented by *X_1_*, *X_2_*, and *X_3_*. The next natural optimization step was to describe the pattern of the *S/L* (or *X_4_*, expressed in g/L) with the purpose to realize more productive processes as requested by industrial applications. In all cases, experimental points are distributed following linear patterns as the *S/L* increases, consequently, linear models with intercept were used to evaluate the responses. The parametric value of the slope (*m*) was used to evaluate the dose response. The increases in the extraction responses are indicated by the positive values, on the other hand, decreases in the extraction efficiency as the *S/L* increases are indicated by the negative values.

### 3.6. Numerical Methods, Statistical Analysis, and Graphical Illustrations

The numerical methods, statistical treatment and calculations, and the graphical illustrations were performed according a methodology described by Prieto & Vázquez [37] and Murado & Prieto [38], using a Microsoft Excel spreadsheet, as follows:

1. The measurement of the coefficients was achieved using the nonlinear least-square (quasi-Newton) method provided by the macro “*Solver*”, by minimization of the sum of the quadratic differences between the observed and model-predicted values.
2. The significance of the coefficients was obtained via “*SolverAid*” macro to determine the parametric confidence intervals. The terms that were not statistically significant (*p*-value > 0.05) were excluded to simplify the model.
3. The model reliability was confirmed by applying the following criteria: a) the Fisher *F*-test (*α* = 0.05) was used to determine the consistency of the constructed models to describe the obtained data; b) the “*SolverStat*” macro was used to make an assessment of the parameter and model prediction uncertainties; c) *R*^²^ was determined to explain the variability proportion of the dependent variable obtained by the model.

Graphical illustrations were generated using DeltaGraph 7.1 (Red Rock Software).

### 3.7. Preparation of the Optimal Extract Rich in Anthocyanin Compounds

The sample (1 g) was added to 20 mL of the solvent (ethanol/water 46%, *v/v*), acidified with 0.25% of citric acid (until pH = 2.5). The extraction was performed at established conditions, temperature (*T* or *X_2_*) of 27 °C and time (*t* or *X_1_*) of 20 min. The obtained extract was evaporated at 35 °C (rotary evaporator Büchi R-210, Flawil, Switzerland) to remove the ethanolic fraction (along with the citric acid) and the aquouse fraction was lyophilized (−47 °C, 0.045 bar; FreeZone 4.5, Labconco, Kansas City, MO, USA) for further analysis and later application in a bakery food product, similar to a donut.

### 3.8. Evaluation of Coloring Potential of the Rich Anthocyanin Extract

The color of the lyophilized extract was measured in three different points using a colorimeter (model CR-400, Konica Minolta Sensing Inc., Tokyo, Japan). The illuminate C was used and a diaphragm aperture of 8 mm that was previously calibrated against a standard white tile. The color space values CIE L* (lightness), a* (greenness/redness), b* (blueness/yellowness) color space values were registered using a data software ‘‘Spectra Magic Nx” (version CM-S100W 2.03.0006, Konica Minolta Company, Japan). For anthocyanin detection, the rich anthocyanin extract (RAE) was evaluated using a HPLC-DAD-ESI/MSn system previously described in Section 3.3.

### 3.9. Bioactivity Evaluation of the Rich Anthocyanin Extract

#### 3.9.1. Cytotoxic and Hepatotoxic Potential

The obtained RAE was evaluated using a procedure previously described by Barros et al. [39] regarding the cytotoxicity assay. Four tumor cell lines were used, such as MCF-7 (breast adenocarcinoma), NCI-H460 (non-small cell lung cancer), HeLa (cervical carcinoma) and HepG2 (hepatocellular carcinoma). On the other hand, the hepatotoxicity was evaluated in a primary non-tumor cell culture (PLP2), where a freshly harvested porcine liver obtained from a local slaughterhouse was used. Ellipticine was used as a positive control, due to its powerful antitumor capacity. The results were expressed in GI_50_ values (sample concentration that inhibits the growth of cells by 50%; μg/mL).

#### 3.9.2. Antimicrobial Activity

To evaluate the antibacterial activity, a methodology previously described by Alves et al. [40] was followed. For the analysis, the activity of RAE (100 mg) was tested against clinical isolates from patients hospitalized in various departments of the Local Health Unit of Bragança and Hospital Center of Trás-os-Montes and Alto-Douro Vila Real, Northeast of Portugal. Thus, five Gram-negative bacteria (*Escherichia coli*, *Klebsiella pneumoniae*, *Morganella morganii*, *Pseudomonas aeruginosa* and *Proteus mirabilis*) and four Gram-positive bacteria (MRSA- methicillin-resistant *Staphylococcus aureus*, *Listeria monocytogenes* and *Enterococcus faecalis*) were used. The microdilution method and the rapid p-iodonitrotetrazolium chloride (INT) colorimetric assay were performed in order to obtain the minimum inhibitory concentrations (MIC—lowest concentration that inhibits the visible bacterial growth) and minimum bactericidal concentration (MBC- lowest concentration of an antibacterial agent required to eradicate a bacteria) and the results were presented in mg/mL. Ampicillin (20 mg/mL), imipenem (1 mg/mL) and vancomycin (1 mg/mL) were used as the positive control.

### 3.10. Incorporation of the Rich Anthocyanin Extract in a Bakery Product

#### 3.10.1. Donuts preparation

**Figure 5:**
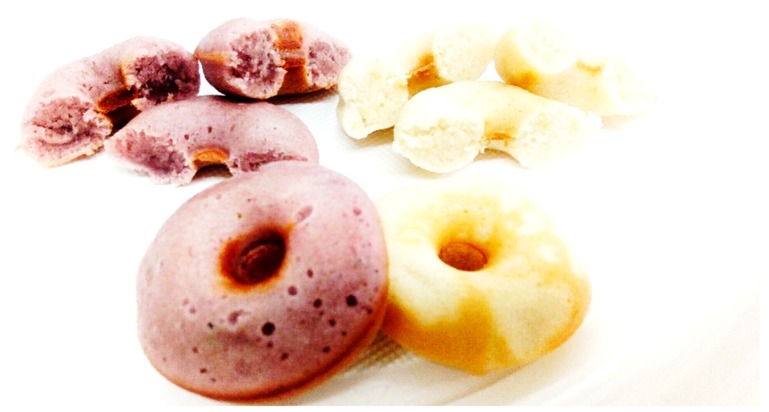
Donuts with an optimal extract rich in anthocyanin compounds (purple donut) and donut controls without the addition of extract (white donuts).

The preparation of the bakery product was made by following these steps: wheat flour (160 g), sugar (90 g) and baking powder (3.5 g) and were combined into a mix, then milk (175 mL) and oil (15 mL) were added while stirring constantly with a hand mixer (Bosch, Munich, Germany) at 450 W during 8 min, until the mixture was thick and creamy. The natural colorant (dry rich anthocyanin extract; 4 g) was dissolved in lemon juice (15 mL) and added to the mixture, while mixed, until it obtained a homogeneous color (near rose/purple). Lemon juice was used to decrease the pH of the mixture and consequently to increase the stability of the anthocyanins. The pH of mixture was measured (Hanna Instruments, Woonsocket, Rhode Island, EUA) in both samples, with and without pigmented extract). The donuts baking machine (Eletronia, model DF-303) was previously heated and greased (using cooking oil), and the mixture was baked for approximately 3 min. Afterwards, the food product was removed (Figure 5).

Four lots of donuts were prepared (8 per lot; 4 for each storage time (T0 = 0 days of storage time (ST) and T3 = 3 days of ST): DRAET0—donuts incorporated with the RAE (T0); DRAET3—donuts incorporated with the RAE (T3); DCT0—donuts without RAE (t = 0 days); and DCT3—donuts without RAE (T3). The obtained donuts were lyophilized (FreeZone 4.5, Labconco, Kansas City, MO, USA), reduced to a fine homogeneous powder and analyzed immediately after preparation and after 3 days of storage time (packed in a sealed plastic bag, protected from light and at room temperature).

#### 3.10.2. Evaluation of the Color Parameters in Donut Samples

The color analysis of the donut samples was performed according to the procedure described in Section 3.7, in order to measure the colorant stability during the donuts’ storage time.

#### 3.10.3. Evaluation of the Nutritional and Chemical Properties of the Donuts

##### Nutritional Value

The protein, fat, carbohydrates and ash content of the donut samples were obtained using AOAC [41] procedures and using methodologies described by Chahdoura et al. [42]. For the crude protein (N × 5.70) a Kjeldahl method (AOAC 978.04) was applied, the ash content was obtained by exposing the sample to incineration at 600 ± 15 °C for 12 h (AOAC 923.03), whereas the crude fat was obtained by using a Soxhlet apparatus with petroleum ether as recycling solvent (AOAC 920.85) and, finally, the total carbohydrate was assessed through difference. To determine the total energy, the following equation was used: Energy (kcal) = 4 × (g protein + g carbohydrates) + 9 × (g fat).

##### Chemical Composition

The chemical composition of the donut samples was determined by assaying the sugars and fatty acids according to methodologies previously described by Barros et al. [39], and analyzed through chromatographic systems, namely, HPLC-RI and GC-FID, respectively. The compounds were characterized by comparison with available standards (standard 47885, Sigma-Aldrich, St. Louis, MO, USA). The content in sugars was expressed in g/100 g of fresh weight, and melezitose (Sigma Chemical Co.; Saint Louis, MO, USA) was used as internal standard in sugars evaluation. The fatty acids concentration was expressed as relative percentages (%) of each fatty acid.

### 3.11. Statistical Analysis

The incorporation and the chemical analysis were performed in triplicate and the results were expressed as mean ± standard deviation (SD). The statistical treatment was performed using Student’s t-test for the color parameters of fruits (fresh and dehydrated) and nutritional and chemical evaluation, in order to examine the presence of significant differences between two samples, at a significance level of 0.05 (SPSS v. 23.0; IBM Corp., Armonk, NY, USA).

## 4. Conclusions

Given that bakery products are produced worldwide and that donuts are highly popular both amongst children and across the general population, replacing artificial ingredients with natural ones is likely to provide many benefits. The present study aimed to establish an optimized extraction procedure for heat assisted extraction (HAE) of anthocyanins from *R. ulmifolius* fruits, using a response surface methodology (RSM). This extraction procedure was applied to achieve the optimal conditions that allowed us to acquire a rich anthocyanin extract (RAE), using the following conditions: t = 20.0 min, T = 56.87 °C and % ethanol = 46.07, in order to obtain a yield of 62.08% with a total anthocyanins content of 33.58 mg AT/g E.

Regarding the coloring potential, the RAE exhibited an intense red-burgundy color, being able to add a pink/lilac color to the donuts after baking while showing itself to be stable along the duration of the storage time, without significant variation of nutritional and chemical values. Furthermore, the RAE also presented bioactive potential that could be explored, especially antimicrobial and cytotoxic properties, within nontoxic concentrations.

In this sense, this study provides a new opportunity to the food industry and a revalorization of this wild species, *Rubus ulmifolius* Schott, which has proved itself to be a rich source of anthocyanin compounds with a high coloring potential that could be used to replace artificial additives.

## Figures and Tables

**Table 1:** Part A shows the experimental RSM results of the *CCCD* with 5 range levels for the HAE optimization of the three main variables involved (X1, X2, and X3). Variables, natural values and ranges are described. Responses comprise the extraction yield (%) and the total content in anthocyanins (AT, mg AT/g E) and the individual content of all major anthocyanins detected (A2 to A5, in mg/g E), in which three replicates were performed for each condition. Part B1 shows the parametric results after fitting the Equation (1) to the responses. Analysis of significance of the parameters (α = 0.05) are presented in coded values. Part B2: shows the variable conditions in natural values that lead to optimal response values for RSM for the responses used.

**A) Experimental Design And Response Results**												
	**Coded and Natural**						**Extract**	**Individual Compounds Content**				**Total**
	*X_1_*	*X_2_*	*X_3_*	*X_1_*:*t* min	*X_2_*:*T* °C	*X_3_*:*S* %	*Yield* %	A2 mg/g E	A3 mg/g E	A4 mg/g E	A5 mg/g E	AT mg/g E
**1**	−1	−1	−1	40.3	34.2	20.3	59.89	10.68	1.58	2.04	1.14	15.44
**2**	−1	−1	1	40.3	34.2	79.7	53.52	9.97	1.83	3.27	1.14	16.21
**3**	−1	1	−1	40.3	75.8	20.3	61.33	15.71	1.95	3.25	1.54	22.45
**4**	−1	1	1	40.3	75.8	79.7	55.83	10.01	1.83	3.35	1.14	16.33
**5**	1	−1	−1	99.7	34.2	20.3	62.55	13.83	2.28	3.24	1.52	20.86
**6**	1	−1	1	99.7	34.2	79.7	52.80	10.76	1.78	3.48	1.17	17.20
**7**	1	1	−1	99.7	75.8	20.3	63.47	15.79	2.31	3.21	1.52	22.83
**8**	1	1	1	99.7	75.8	79.7	56.12	9.53	1.75	3.23	1.14	15.65
**9**	1.68	0	0	120	55	50	66.06	24.06	2.94	6.41	2.13	35.55
**10**	−1.68	0	0	20	55	50	60.88	23.63	1.85	6.33	2.16	33.97
**11**	0	−1.68	0	70	20	50	59.06	4.36	1.08	1.19	0.86	7.50
**12**	0	1.68	0	70	90	50	63.31	8.04	1.33	1.75	1.06	12.18
**13**	0	0	−1.68	70	55	0	61.59	5.95	1.25	1.64	0.97	9.82
**14**	0	0	1.68	70	55	100	44.82	9.56	1.64	2.95	1.03	15.18
**15**	−1.68	−1.68	−1.68	20	20	0	65.19	10.55	1.73	2.20	1.23	15.72
**16**	−1.68	−1.68	1.68	20	20	100	40.44	5.89	1.37	1.96	0.87	10.08
**17**	−1.68	1.68	−1.68	20	90	0	59.97	14.02	2.01	3.21	1.56	20.81
**18**	−1.68	1.68	1.68	20	90	100	53.96	9.90	1.60	3.09	0.56	15.15
**19**	1.68	−1.68	−1.68	120	20	0	65.99	2.91	1.03	1.15	0.79	5.87
**20**	1.68	−1.68	1.68	120	20	100	42.16	4.43	1.69	3.01	1.11	10.24
**21**	1.68	1.68	−1.68	120	90	0	74.03	8.97	1.54	1.98	1.11	13.60
**22**	1.68	1.68	1.68	120	90	100	50.83	9.15	1.53	2.86	1.00	14.54
**23**	0	0	0	70	55	50	61.33	21.33	2.80	5.15	2.00	31.27
**24**	0	0	0	70	55	50	57.47	22.34	3.00	6.45	2.13	33.92
**25**	0	0	0	70	55	50	58.93	22.34	2.69	5.37	1.86	32.26
**26**	0	0	0	70	55	50	57.47	22.60	2.73	5.45	1.94	32.73
**27**	0	0	0	70	55	50	58.56	21.09	2.68	5.62	1.87	31.26
**28**	0	0	0	70	55	50	56.64	22.63	3.09	5.98	1.94	33.65
**B) Parametric Values and Optimal Variable Conditions**												
							**Extract**	**Individual Compounds Content**				**Total**
							*Yield*	A2	A3	A4	A5	AT
**B1) Parametric Information and Statistical Information**												
Intercept				*b_0_*			58.312 ± 0.834	22.091 ± 0.654	2.837 ± 0.105	5.587 ± 0.177	1.934 ± 0.054	32.502 ± 0.868
Linear effect				*b_1_*			0.983 ± 0.344	−0.573 ± 0.325	ns	ns	ns	−0.569 ± 0.432
				*b_2_*			1.577 ± 0.344	1.177 ± 0.325	0.062 ± 0.043	0.185 ± 0.073	0.030 ± 0.022	1.454 ± 0.432
				*b_3_*			−5.182 ± 0.344	−0.595 ± 0.325	ns	0.215 ± 0.073	−0.081 ± 0.022	−0.475 ± 0.432
Quadratic effect				*b_11_*			1.711 ± 0.584	ns	−0.150 ± 0.073	0.170 ± 0.124	0.046 ± 0.038	ns
				*b_22_*			0.902 ± 0.584	−5.938 ± 0.539	−0.571 ± 0.073	−1.563 ± 0.124	−0.372 ± 0.038	−8.458 ± 0.715
				*b_33_*			−1.918 ± 0.584	−5.387 ± 0.539	−0.487 ± 0.073	−1.271 ± 0.124	−0.358 ± 0.038	−7.518 ± 0.715
Interactive effect				*b_12_*			0.337 ± 0.244	ns	ns	−0.079 ± 0.052	ns	ns
				*b_13_*			−0.712 ± 0.244	0.371 ± 0.231	0.039 ± 0.031	0.107 ± 0.052	0.058 ± 0.016	0.576 ± 0.306
				*b_23_*			0.806 ± 0.244	ns	−0.034 ± 0.031	−0.053 ± 0.052	−0.048 ± 0.016	ns
Additional complex effect				*b_1122_*			ns	ns	ns	ns	ns	ns
				*b_1133_*			ns	ns	ns	ns	ns	ns
				*b_2233_*			−0.469 ± 0.363	2.256 ± 0.259	0.268 ± 0.046	0.542 ± 0.077	0.128 ± 0.024	3.220 ± 0.344
***Statistics (R^2^)***							***0.9336***	***0.9276***	***0.8908***	***0.9411***	***0.9294***	***0.9330***
**B2) Optimal Variable Conditions for Response Maximization**												
Individual optimal conditions				Time			120.00 ± 3.60	20.00 ± 0.20	69.99 ± 3.50	20.00 ± 1.60	20.00 ± 1.00	20.00 ± 1.00
				Temperature			90.00 ± 9.00	57.07 ± 0.57	56.13 ± 2.25	57.11 ± 4.00	56.20 ± 3.37	56.79 ± 1.14
				Solvent			26.97 ± 2.16	46.62 ± 0.93	49.94 ± 4.49	50.34 ± 5.03	42.42 ± 2.12	47.14 ± 0.94
				Response			72.91 ± 3.54	23.18 ± 3.54	2.84 ± 3.54	6.09 ± 3.54	2.09 ± 3.54	33.59 ± 3.54
Global optimal conditions				Time			20.00 ± 0.60					
				Temperature			56.87 ± 3.41					
				Solvent			46.07 ± 3.69					
				Response			62.08 ± 3.54	23.18 ± 3.54	2.42 ± 3.54	6.06 ± 3.54	2.08 ± 3.54	33.58 ± 3.54

**Table 2:** Experimental domain of independent variables in the *CCCD* with 5 range levels.

Coded Values	Natural Values		
	t (min)	T (°C)	S (%)
−1.68	20	20	0
−1	40.3	37.2	20.3
0	70	55	50
+1	99.7	72.8	79.7
+1.68	120	90	100

**Table 3:** Quantification of anthocyanin compounds in the extract and color evaluation (CIE L*, a*, b*).

Anthocyanins (mg AT/g E)	L*	a*	b*	Conversion Color to RGB Values
33.2 ± 0.8	24.8 ± 0.1	31.7 ± 0.5	7.6 ± 0.2	

AT—total anthocyanins; L*—lightness; a* (greenness/redness), b* (blueness/yellowness); the results are presented as the mean ± SD.

**Table 4:** Results of antimicrobial and cytotoxicity activities of the rich anthocyanin extract.

**A) Antimicrobial Activity**									
		**Extract**		**Ampicillin**		**Imipenem**		**Vancomycin**	
		**MIC**	**MBC**	**MIC**	**MBC**	**MIC**	**MBC**	**MIC**	**MBC**
	**Gram-Negative Bacteria (mg/mL)**								
	Escherichia coli	5	>20	<0.15	<0,.5	<0.0078	<0.0078	n.t.	n.t.
	Klebsiella pneumoniae	20	>20	10	20	<0.0078	<0.0078	n.t.	n.t.
	Morganella morganii	2.5	>20	20	>20	<0.0078	<0.0078	n.t.	n.t.
	Proteus mirabilis	10	>20	<0.15	<0.15	<0.0078	<0.0078	n.t.	n.t.
	Pseudomonas aeruginosa	>20	>20	>20	>20	0.5	1	n.t.	n.t.
	**Gram-Positive Bacteria (mg/mL)**								
	Enterococcus faecalis	10	>20	<0.15	<0.15	n.t.	n.t.	<0.0078	<0.0078
	Listeria monocytogenes	5	>20	<0.15	<0.15	<0.0078	<0.0078	n.t.	n.t.
	MRSA	2.5	>20	<0.15	<0.15	n.t.	n.t.	0.25	0.5
**B) Citotoxicity Activity**									
		**Rich extract**		**Ellipticin**					
	**Cytotoxicity Activity (GI_50_, µg/mL)**								
	HeLa	301 ± 19		1.91 ± 0.06					
	NCI H460	337 ± 11		1.0 ± 0.1					
	MCF7	328 ± 13		0.91 ± 0.04					
	HepG2	286 ± 13		1.1 ± 0.2					
	**Hepatotoxicity (GI_50_, µg/mL)**								
	PLP2	>400		3.2 ± 0.7					

MRSA—methicillin-resistant Staphylococcus aureus; HeLa—cervical carcinoma cell culture; NCI H460—non-small cell lung carcinoma; MCF7—breast adenocarcinoma; HepG2—hepatocellular carcinoma cell culture; GI_50_—values correspond to the sample concentration achieving 50% of growth inhibition in human tumor cell lines or in liver primary culture PLP2; MBC—minimum bactericidal concentration; MIC—minimum inhibitory concentration. The results are presented as the mean ± SD.

**Table 5:** Color parameters (CIE L*, a*, b*), nutritional value, free sugars (g/100 g fw) and fatty acids (%) composition of the donut samples during storage time (T0 and T3 days).

	DCT0	DRAET0	*p*-Value	DCT3	DRAET3	*p*-Value
**Color parameters**						
L*	76 ± 1	57,5 ± 0.4	<0,01	77.0 ± 0.3	57.0 ± 0.3	<0.01
a*	−0.1 ± 0.2	10.8 ± 0.4	<0,01	0.03 ± 0.0	10.2 ± 0.1	<0.01
b*	19.7 ± 0.3	10.9 ± 0.5	<0,01	20.0 ± 0.3	10.4 ± 0.6	<0.01
**Nutritional value**						
Moisture (g/100 g fw)	22.13 ± 0.8	28.02 ± 0.1	< 0.01	22.35 ± 0.3	27.19 ± 0.5	< 0.01
Proteins (g/100 g fw)	6.9 ± 0.1	6.936 ± 0.003	0.134	6.8 ± 0.2	6.82 ± 0.04	0.165
Ash (g/100 g fw)	0.97 ± 0.02	0.96 ± 0.02	0.334	0.97 ± 0.02	0.97 ± 0.02	0.430
Fat (g/100 g fw)	5.0 ± 0.1	4.6 ± 0.1	0.133	4.7 ± 0.1	4.9 ± 0.1	0.058
Carbohydrates (g/100 g fw)	64.91 ± 0.04	59.5 ± 0.1	< 0.01	65.2 ± 0.1	60.10 ± 0.03	< 0.01
Energy (kcal/100 g fw)	332.8 ± 0.4	307.2 ± 0.4	< 0.01	330.1 ± 0.8	311.9 ± 0.5	< 0.01
pH	6.40 ± 0.14	5.23 ± 0.02	-	-	-	-
**Free sugars (g/100 g fw)**						
Fructose	0.07 ± 0.01	0.63 ± 0.04	<0.01	0.07 ± 0.01	0.58 ± 0.01	<0.01
Glucose	0.06 ± 0.01	0.51 ± 0.04	<0.01	0.07 ± 0.01	0.53 ± 0.04	<0.01
Sucrose	17.5 ± 0.6	16.2 ± 0.3	<0.01	17.6 ± 0.6	15.2 ± 0.3	<0.01
Threhalose	1.6 ± 0.1	1.92 ± 0.04	<0.01	1.7 ± 0.1	1.78 ± 0.1	0.004
***Total***	19.2 ± 0.7	19.3 ± 0.2	0.619	19.4 ± 0.7	18.1 ± 0.3	<0.01
**Fatty acids (%)**						
C6:0	0.28 ± 0.01	0.32 ± 0.01	0.367	0.29 ± 0.01	0.337 ± 0.001	0.609
C8:0	0.18 ± 0.01	0.198 ± 0.004	0.289	0.179 ± 0.002	0.205 ± 0.001	0.275
C10:0	0.41 ± 0.02	0.465 ± 0.003	0.163	0.41 ± 0.01	0.482 ± 0.005	0.287
C11:0	0.019 ± 0.001	0.019 ± 0.001	1.000	0.020 ± 0.001	0.021 ± 0.001	0.998
C12:0	0.49 ± 0.02	0.585 ± 0.002	0.144	0.48 ± 0.01	0.56 ± 0.01	0.743
C13:0	0.022 ± 0.001	0.022 ± 0.001	1.000	0.019 ± 0.001	0.023 ± 0.001	0.432
C14:0	1.4 ± 0.1	1.589 ± 0.003	0.132	1.40 ± 0.01	1.61 ± 0.01	0.338
C14:1	0.12 ± 0.01	0.131 ± 0.001	0.165	0.114 ± 0.001	0.134 ± 0.001	0.070
C15:0	0.17 ± 0.01	0.183 ± 0.003	0.367	0.17 ± 0.1	0.187 ± 0.004	0.559
C16:0	10.4 ± 0.3	10.8 ± 0.1	0.215	10.39 ± 0.07	10.87 ± 0.04	0.537
C16:1	0.29 ± 0.01	0.314 ± 0.003	0.498	0.291 ± 0.001	0.312 ± 0.001	0.898
C17:0	0.13 ± 0.01	0.14 ± 0.01	0.639	0.130 ± 0.001	0.140 ± 0.004	0.116
C17:1	0.039 ± 0.001	0.045 ± 0.001	0.116	0.039 ± 0.001	0.045 ± 0.002	0.116
C18:1n9t	3.7 ± 0.1	3.83 ± 0.01	0.155	3.71 ± 0.02	3.76 ± 0.01	0.609
C18:1n9c	19 ± 1	18.4 ± 0.6	0.373	18.8 ± 0.5	19.25 ± 0.03	0.133
C18:2n6	62 ± 1	60.9 ± 0.5	0.471	61.6 ± 0.3	60.0 ± 0.1	0.236
C18:3n3	0.530 ± 0.001	0.72 ± 0.01	0.242	0.508 ± 0.001	0.716 ± 0.003	0.219
C20:0	0.219 ± 0.001	0.221 ± 0.001	0.116	0.22 ± 0.01	0.219 ± 0.001	0.151
C20:1	0.158 ± 0.002	0.161 ± 0.002	0.897	0.153 ± 0.001	0.15 ± 0.01	0.152
C20:2	0.137 ± 0,001	0.135 ± 0.001	0.921	0.149 ± 0.004	0.127 ± 0.004	0.811
C20:3n3	0.024 ± 0.002	0.030 ± 0.003	0.710	0.03 ± 0.01	0.037 ± 0.001	0.422
C22:0	0.61 ± 0.01	0.589 ± 0.004	0.377	0.613 ± 0.001	0.571 ± 0.001	0.989
C24:0	0.20 ± 0.01	0.239 ± 0.003	0.197	0.27 ± 0.02	0.20 ± 0.01	0.202
**SFA**	14.5 ± 0.4	15.4 ± 0.1	0.231	14.6 ± 0.1	15.4 ± 0.1	0.458
**MUFA**	23 ± 1	22.8 ± 0.6	0.387	23.1 ± 0.4	23.66 ± 0.02	0.128
**PUFA**	62 ± 1	61.7 ± 0.5	0.467	62.3 ± 0.3	60.9 ± 0.1	0.253

DCT0—donut without RAE at T0; DCT3—donut without RAE at T3; DRAET0—donut with RAE at T0; DRAE—donut with RAE at T3. L*—lightness; a* (greenness/redness), b* (blueness/yellowness). SFA; saturated fatty acids MUFA: monounsaturated fatty acids; PUFA: polyunsaturated fatty acids. Caproic *acid* (C6:0); Caprylic *acid (C8:0)*; Capric *acid (C10:0)*; *Undecanoic acid (C11:0)*; Dodecanoic *acid*, *(C12:0)*, Tridecanoic acid (C13:0); Myristic acid (C14:0); Myristoleic acid (C14:1); Pentadecanoic acid (C15:0); Palmitic acid (C16:0); Palmitoleic acid (C16:1); Heptadecanoic acid (C17:0); Heptadecanoic acid (C17:1); *cis*-Oleic acid- (C18:1n9c); *trans-*Oleic acid (C18:1n9t); Linoleic acid (C18:2n6); α-Linolenic acid (C18:3n3); Stearic acid (C20:0); Eicosenoic acid (C20:1); Eicosadienoic acid (C20:2); Eicosatrienoic acid (C20:3n3); Behenic acid (C22:0); Lignoceric acid (C24:0). Results are presented as mean ± SD.
